# Extracellular-to-total body water ratio is associated with comorbidity and cardiorespiratory fitness in older adults with post-COVID-19 syndrome

**DOI:** 10.3389/fnut.2026.1715783

**Published:** 2026-02-11

**Authors:** Eulogio Pleguezuelos, Amin Del Carmen, Sergio Sánchez-Nuño, Noemí Serra-Payá, Eva Moreno, Lorena Molina-Raya, Carmen Jerez-Molina, Montserrat Girabent Farrés, Jorge Castizo-Olier, Ainoa Biurrun-Garrido, Xavier Viñals, Mateu Serra-Prat, Manuel Vicente Garnacho-Castaño

**Affiliations:** 1 Department of Physical Medicine and Rehabilitation, Mataró Hospital, Mataró, Barcelona, Spain; 2 DAFNiS Research Group, Pain, Physical Activity, Nutrition and Health, Campus Docent Sant Joan de Déu, Universitat de Vic-Universitat Central de Catalunya, Sant Boi de Llobregat, Barcelona, Spain; 3 Servicio de Medicina Física y Rehabilitación, Hospital General de Hospitalet, L'Hospitalet de Llobregat, Barcelona, Spain; 4 Departament de Salut Publica, Salut Mental i Materno-Infantil, Facultat d'Infermeria, Universitat de Barcelona, Barcelona, Spain; 5 Unidad de Investigación Consorci Sanitari del Maresme, Mataró, Barcelona, Spain; 6 CienciaSalud Integral, Palma de Mallorca, Spain; 7 Facultad de Ciencias de la Salud, Universidad Internacional de Valencia (VIU), Valencia, Spain

**Keywords:** aging, bioelectrical impedance analysis, cardiopulmonary exercise testing, hydration, isokinetic test, long COVID-19, muscular fitness, peak oxygen uptake

## Abstract

**Background:**

Post-coronavirus disease 2019 (post-COVID-19) syndrome is associated with persistent impairments in physical fitness and altered body composition, particularly in older adults. The extracellular-to-total body water (ECW/TBW) ratio has been linked to poor outcomes in clinical populations. However, its association with cardiorespiratory fitness (CRF) and muscular fitness (MF) in older adults with post-COVID-19 syndrome remains unclear. This study aimed to examine the associations between ECW/TBW ratio, CRF, MF, and other variables in this population.

**Methods:**

A cross-sectional study was conducted in 71 older adults with post-COVID-19 syndrome. Hydration status and body composition were assessed using bioelectrical impedance analysis (BIA). CRF was evaluated by cardiopulmonary exercise testing (CPET; peak oxygen uptake, VO_2_peak), and MF was assessed using isokinetic and functional performance tests. Associations between ECW/TBW ratio, fitness outcomes, and other variables were analyzed through multi-variate linear regression models adjusted for age and sex. Results: Higher ECW/TBW ratio was significantly associated with lower VO_2_peak (β = −0.010, *p* = 0.048) and greater comorbidity burden (β = 0.003, *p* = 0.002). No significant associations were observed between ECW/TBW ratio and MF variables (*p* > 0.05).

**Conclusions:**

The ECW/TBW ratio is independently associated with comorbidity burden and CRF, but not with MF, in older adults with post-COVID-19 syndrome. The Charlson Comorbidity Index emerged as the strongest determinant of ECW/TBW ratio. These findings highlight the potential relevance of integrating hydration monitoring and CRF assessment into rehabilitation strategies, and support further investigation of their role in the clinical management of older adults with post-COVID-19 syndrome.

## 1 Introduction

Post-coronavirus disease 2019 (post-COVID-19) syndrome, or long COVID, remains a major clinical challenge, particularly in older adults. Up to one year after hospital discharge, many patients continue to present with persistent symptoms affecting multiple systems, including fatigue, dyspnea, chest tightness, palpitations, dizziness, headache, gastrointestinal disturbances, and psychological distress (1, 2). Older adults are especially vulnerable, as pre-existing comorbidities and age-related physiological changes exacerbate infection severity and increase the risk of prolonged post-acute sequelae (3). In this context, cumulative comorbidity burden—commonly quantified using the Charlson Comorbidity Index—has been consistently associated with a higher risk of adverse clinical outcomes in COVID-19. Beyond its prognostic value during the acute phase, comorbidity burden may also influence post-acute recovery trajectories and long-term physiological vulnerability in older adults (4). Despite growing recognition of long COVID, its long-term consequences and underlying mechanisms are still poorly understood.

Fatigue, reduced exercise tolerance, and impairments in both cardiorespiratory fitness (CRF) and muscular fitness (MF) are among the most frequent and disabling complications of post-COVID-19 syndrome, particularly in older adults (5–8). These limitations substantially diminish quality of life and contribute to greater functional dependency (9).

CRF, commonly assessed by maximal or peak oxygen uptake (VO_2_max or VO_2_peak), is a robust predictor of health, mortality and longevity (10, 11). However, mounting evidence shows that CRF is frequently and substantially impaired in patients recovering from COVID-19 (6, 12, 13). MF, encompassing muscle strength and body composition, is equally vital for maintaining or improving muscle mass and increasing muscle strength, thereby enhancing functional capacity and independence in older adults (14, 15). Evidence indicates post-COVID-19 reductions in VO_2_peak, ventilatory efficiency, muscle strength, and skeletal muscle mass in older people (6). Despite the complementary contributions of CRF and MF to overall physical fitness, their respective roles in post-COVID-19 recovery remain poorly defined (16, 17), leaving an important gap in knowledge. Understanding the physiological and functional changes associated with long COVID is critical for developing targeted and effective rehabilitation strategies for this vulnerable population (8).

In addition to fitness impairments, nutritional and hydration deficits, altered body composition and severe muscle weakness are also prevalent in this population, particularly among patients who were bedridden during the acute phase (18–20). These clinical issues underscore the importance of comprehensive evaluation of body composition and hydration status, which enables early identification of malnutrition, fluid imbalance, and muscle mass loss, thereby informing targeted nutritional and physical rehabilitation interventions (20, 21). Bioelectrical impedance analysis (BIA) is a noninvasive, reliable, and validated method for assessing body composition and fluid distribution in clinical settings (22, 23), including in older adults (24–28), and has been applied in studies of patients with post–COVID-19 syndrome (29, 30).

Among the parameters obtained from BIA, the extracellular water-to-total body water (ECW/TBW) ratio stands out for its clinical relevance. This index provides an integrated measure of hydration status by quantifying the proportion of extracellular water relative to total body water, thereby reflecting potential fluid imbalances. Beyond its role as a marker of hydration status, the ECW/TBW ratio is increasingly recognized as a multifactorial biomarker reflecting broader pathophysiological processes, including loss of cellular mass, alterations in body composition, and systemic inflammation. An elevated ECW/TBW ratio may reflect a relative expansion of extracellular water together with a reduction in intracellular water, a pattern commonly observed in sarcopenia, chronic inflammatory states, and advanced multimorbidity (31–34). In this context, ECW/TBW may serve as an indirect indicator of muscle quality and cellular integrity rather than fluid imbalance alone.

The ECW/TBW ratio has been widely applied in various clinical contexts (35, 36), including in patients with COVID-19 (30). Notably, Cornejo-Pareja et al. (30) demonstrated that overhydration, as indicated by an elevated ECW/TBW ratio, was a significant independent predictor of 90-day mortality in patients with COVID-19. Furthermore, Gryglewska-Wawrzak et al. (37) identified an association between increased left ventricular volumes and fat content with reduced VO_2_peak 15 months after recovery from COVID-19; however, no significant relationship has been found between CRF and the ECW/TBW ratio. These findings suggest that the relationship between fluid-related parameters and CRF may be influenced by population-specific characteristics. In this regard, the cohort examined by Gryglewska-Wawrzak et al. (37) comprised a younger and clinically more heterogeneous population, with a lower comorbidity burden and without a specific focus on older adults referred to rehabilitation, which may partly explain the absence of an association with ECW/TBW.

Consequently, the higher multimorbidity burden, reduced CRF and MF, and increased susceptibility to disturbances in fluid homeostasis that characterize older adults with post-COVID-19 syndrome (2–8, 30) provide a compelling rationale for examining the relationship between ECW/TBW and measures of CRF and MF in this specific population. Importantly, the potential association between the ECW/TBW ratio and muscle strength has not yet been explored. Exploring a potential association between the ECW/TBW ratio and either CRF or MF may provide novel insights into the physiological mechanisms underlying functional decline and vulnerability in older adults with post-COVID-19 syndrome.

The present exploratory study aimed to examine the associations between the ECW/TBW ratio and CRF, MF and clinical burden, as assessed by the Charlson Comorbidity Index, in older patients with post-COVID-19 syndrome. Based on previous evidence, we explored whether the ECW/TBW ratio was associated with CRF and MF.

## 2 Materials and methods

### 2.1 Study design

This observational cross-sectional study was conducted between January 2023 and January 2024 at the Department of Physical Medicine and Rehabilitation, Hospital de Mataró (Spain). The study was designed to evaluate the association between CRF, MF, and body water content—specifically the extracellular water-to-total body water ratio (ECW/TBW)—in older adults with post-COVID-19 syndrome.

The study adhered to the Declaration of Helsinki and was approved by the Clinical Research Ethics Committee of the Health Care Consortium (CEIm; code 16/21). All participants provided written informed consent prior to enrolment. Reporting follows STROBE recommendations (38).

### 2.2 Participants

A total of 80 older adults (≥60 years) with a clinical diagnosis of post-COVID-19 syndrome were consecutively recruited between January 2023 and January 2024. Participants were recruited from older adults with persistent post-COVID-19 symptoms who were referred from hospital outpatient clinics to the rehabilitation service of the same hospital for further evaluation and management. Post-COVID-19 syndrome was defined according to the World Health Organization (WHO) criteria as the presence of symptoms occurring usually within 3 months from the onset of a confirmed SARS-CoV-2 infection, lasting for at least 2 months, and not explained by an alternative diagnosis (39).

The diagnosis was established by a physician based on clinical evaluation and medical records, including laboratory-confirmed SARS-CoV-2 infection (RT-PCR) and persistent symptoms. The presence of post-COVID-19 symptoms (e.g., fatigue, dyspnea, muscle weakness) was systematically assessed during clinical evaluation using standardized medical history and patient self-report.

All clinical and functional assessments were completed within 14 days of recruitment. On average, participants were evaluated 5.8 ± 2.7 months after the acute SARS-CoV-2 infection (range: 3.1–8.5 months). Data processing and statistical analyses were conducted between April and July 2024, after the end of the recruitment and data collection period.

Patient eligibility was determined through a coordinated review by the study coordinator and a designated hospital researcher. In cases of uncertainty, a physician from the research team evaluated the case to ensure adherence to inclusion criteria and reduce the risk of selection bias. Prior to data collection, all research staff participated in a structured training program led by the study coordinator to guarantee uniform application of inclusion criteria and standardized data collection procedures.

**Figure 1:**
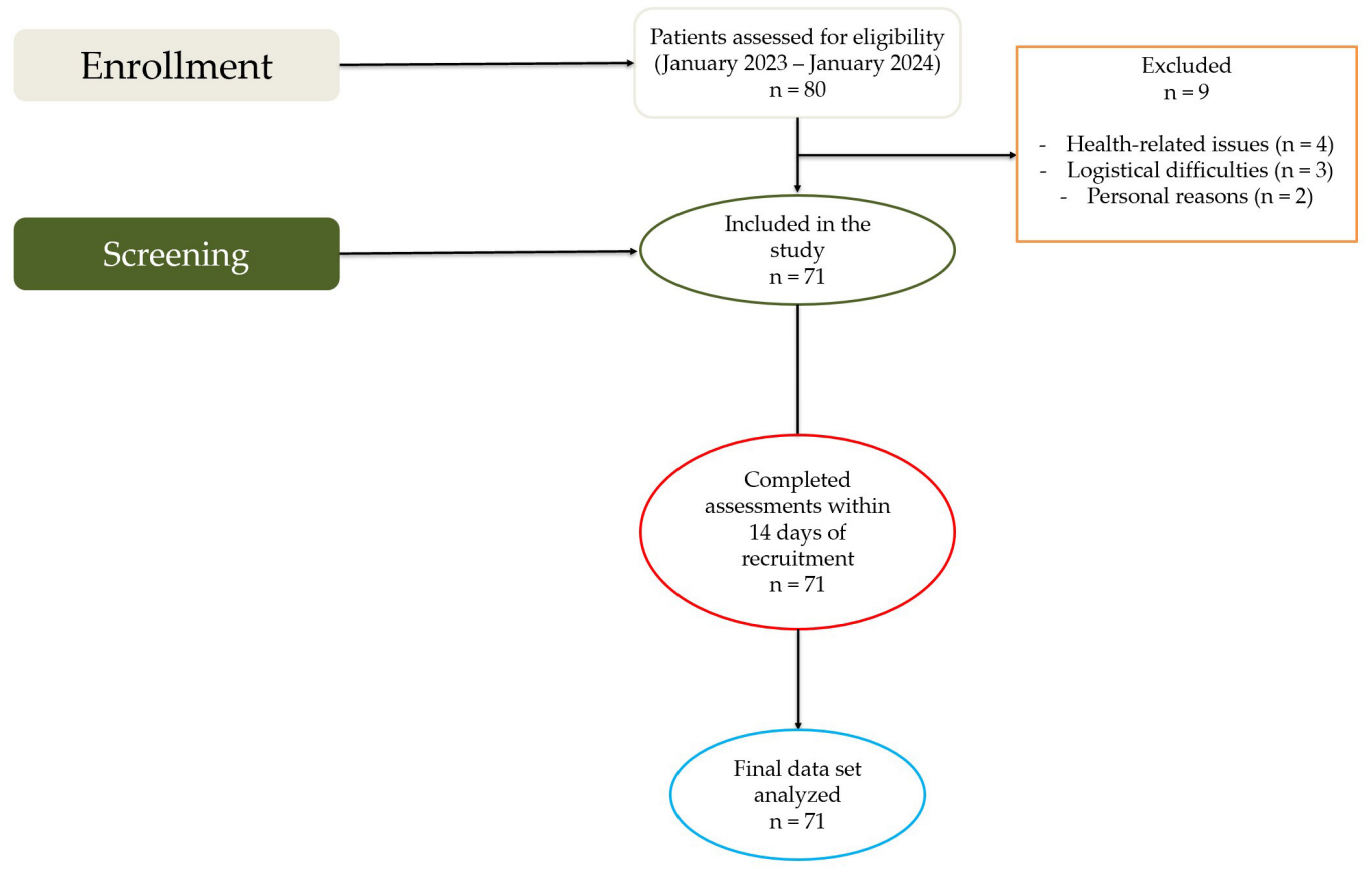
Flow diagram of participant recruitment, screening, inclusion, and analysis for older adults with post-COVID-19 syndrome.

Inclusion criteria were: age ≥60 years, laboratory-confirmed SARS-CoV-2 infection (via RT-PCR), and persistence of post-COVID-19 symptoms for >3 months after acute infection. Exclusion criteria included lack of informed consent, presence of severe neurological, oncological, neuromuscular, or orthopedic disorders, any health-related limitation that prevented participation in physical testing or evaluation procedures, current smoking or alcohol consumption and any factor limiting reliable assessment. Participants excluded due to smoking (*n* = 4) or alcohol (*n* = 1) consumption were identified during the initial eligibility screening and were not included in the enrollment flow. Of 80 screen-positive individuals, nine were excluded due to health- or logistics-related reasons, yielding a final sample of 71 for analysis (Figure 1).

Relevant clinical data were collected for all participants, including adjusted morbidity group classification, comorbidity burden assessed using the Charlson Comorbidity Index, and severity of illness during hospitalization, assessed via the Acute Physiology and Chronic Health Disease Classification System II score (APACHE II) (40–42). The Charlson Comorbidity Index was calculated based on documented medical diagnoses according to established criteria and used as a summary measure of multimorbidity (42–44). The most common symptoms in this population were persistent fatigue, muscle weakness, and dyspnea.

### 2.3 Data collection and outcome measures

Assessments were performed over three visits within 7–14 days, scheduled at the same time of day to minimize diurnal variation. Examiners conducting CRF/MF testing were blinded to BIA results, and the BIA operator was blinded to CRF/MF data.

#### 2.3.1 Cardiorespiratory fitness

CRF was assessed through incremental cardiopulmonary exercise testing (CPET) on an electromagnetically braked cycle ergometer (Ergoline900S, Ergoline GmbH, Germany), under physician supervision as in previous studies (6, 13). Gas exchange was analyzed using an open-circuit metabolic system (Ergostik, Geratherm Respiratory, Germany), calibrated before each test (3-L syringe for volume; certified reference gases for O_2_/CO_2_). Participants were continuously monitored via 12-lead ECG and non-invasive blood pressure. An individualized ramp protocol (5–10 W·min^−1^) was implemented to ensure a progressive increase in workload while maintaining a cadence of 50–70 rpm. Exercise continued until volitional exhaustion. Variables recorded included relative (ml·kg^−1^·min^−1^) and absolute and (L·min^−1^) VO_2_peak, ventilatory efficiency indices (VE·VO_2_^−1^, VE·VCO_2_^−1^), minute ventilation (VE), respiratory exchange ratio (RER), end-tidal partial pressures of O_2_ and CO_2_ (PetO_2_, PetCO_2_), heart rate, power output performance, and time to exhaustion were recorded.

#### 2.3.2 Muscular fitness

Isokinetic knee extensor/flexor strength was assessed using a dynamometer (Biodex System; Software v4.x; Biodex Medical Systems, Shirley, NY, USA) (6). Participants were seated with hip flexion ~85–90°; the dynamometer axis was aligned with the lateral femoral epicondyle; pelvis, thigh, and torso were stabilized with straps. After familiarization (ROM set to 80°, 5 reps at 60°/s, 20-s rest; 5 reps at 180°/s, 15-s rest), participants performed two sets of 5 concentric knee extensions/flexions at 60°/s and 180°/s in randomized order, with 60-s inter-set recovery. Standardized verbal encouragement was provided. The highest peak torque (N·m) and peak power (W) across repetitions at each velocity were used for analysis.

In addition to the isokinetic evaluation, functional MF was assessed on a separate day from isokinetic and cardiorespiratory tests to avoid fatigue carry-over, in the following sequence with standardized tests: Four Square Step Test (FSST), Timed Up and Go (TUG) test, and the 30-Second Sit-to-Stand test (45–47). FSST was used to assess dynamic balance and agility. Participants completed three valid trials, with the best time (seconds) recorded. A 1-min rest was provided between trials. TUG measured functional mobility. One time trial was completed after a 1-min recovery period. 30-Second Sit-to-Stand test assessed lower limb strength and endurance. Participants performed as many full stands as possible in 30 s, following a brief familiarization trial. A 5-min rest was given between tests. All tests were administered by trained physiotherapists under standardized instructions and safety procedures.

Body composition and hydration status were assessed using bioelectrical impedance analysis (BIA) with the InBody S10^®^ system (InBody Co., Ltd., Biospace, California, USA) as in a previous study (6). Participants were assessed supine after a ≥10-min rest in a thermoneutral room, fasting ≥4 h, having avoided strenuous exercise for 24 h, alcohol/caffeine for 12–24 h, and after voiding. Electrodes were placed according to the manufacturer's protocol.

Variables obtained included body weight (kg), fat mass (kg, %), fat-free mass (kg), skeletal muscle mass (kg), total body water (TBW, L, %), extracellular water (ECW, L, %TBW), intracellular water (ICW, L, %TBW), and ECW/TBW ratio (as the primary outcome). BIA was performed on a day separate from the rest of the tests to avoid acute fluid shifts related to exercise. An ECW/TBW ratio ≥0.40 was used to define elevated extracellular water, based on previous literature indicating that this threshold reflects altered fluid distribution and relative overhydration in older and clinical. populations assessed by bioelectrical impedance analysis. This cut-off has been widely applied in studies involving older adults and patients with chronic clinical conditions (33, 48).

### 2.4 Statistical analysis

All statistical analyses were performed using IBM SPSS Statistics (version 28.0; IBM Corp., Armonk, NY, USA). Data were first examined for normality using the Kolmogorov–Smirnov test. Continuous variables are expressed as mean ± standard deviation (SD) for normally distributed data, and categorical variables as frequencies and percentages. To further examine the relationship between ECW/TBW and descriptive, clinical, and physical fitness variables, univariate linear regression analyses were first performed, followed by multivariate models including variables with *p* < 0.10 in univariate testing. To limit model complexity and reduce the risk of overfitting given the sample size, only a restricted number of predictors were included in each multivariate model, ensuring an adequate events-per-variable ratio. Model fit was assessed using the coefficient of determination (*R*^2^ and adjusted *R*^2^), and multicollinearity was evaluated using variance inflation factors (VIF). A VIF > 5 was considered indicative of potentially problematic multicollinearity (49). Regression coefficients (β), 95% confidence intervals (CIs), and *p*-values were reported. Between-group comparisons by sex and age group (≤ 64 vs. >64 years) were conducted using independent samples *t*-tests for continuous variables. Receiver operating characteristic (ROC) curve analyses were performed to assess the discriminatory ability of log-transformed VO_2_peak and the Charlson Comorbidity Index to identify individuals with elevated ECW/TBW ratio (≥0.40). The area under the curve (AUC) with 95% confidence intervals was calculated for each predictor, with values interpreted as poor (< 0.60), fair (0.60–0.70), good (0.70–0.80), or excellent (>0.80). When a clinically meaningful cut-off could be defined, sensitivity and specificity were derived using the Youden index.

Missing data were handled using complete-case analyses, as the proportion of missing values was low and missingness was considered to be random. No formal sample size calculation was performed due to the exploratory nature of the study. Potential sources of bias, including selection bias and residual confounding, were considered in the study design. A two-tailed *p*-value < 0.05 was considered statistically significant for all tests.

## 3 Results

Descriptive and clinical data of older patients with COVID-19 are presented in Table 1. The association between ECW/TBW ratio and various descriptive, clinical, and CRF (Table 2) and MF (Table 3) variables, was analyzed using univariate and multivariate linear regression models.

**Table 1:** Descriptive, anthropometric, and clinical characteristics of older adults with post-COVID-19 syndrome (*n* = 71).

**Variable**	**Women**	**Men**	***p*-Value**	** ≤ 64 years**	**>64 years**	***p*-Value**
*N* (%)	27 (38)	44 (62)		35 (49.29)	36 (50.71)	
Age (years)	65.04 (5.40)	65.18 (4.85)	0.907	61.09 (1.15)	69.06 (4.13)	< 0.001
Height (meters)	1.57 (0.06)	1.71 (0.06)	< 0.001	1.66 (0.09)	1.66 (0.09)	0.763
Weight (kg)	74.71 (13.22)	85.06 (16.09)	0.006	83.25 (15.42)	79.06 (16.09)	0.267
BMI (kg·m^−2^)	30.16 (4.80)	28.96 (4.90)	0.187	30.14 (4.98)	28.71 (4.70)	0.280
Hospitalization (%)	14 (19.70%)	40 (56.30%)	< 0.001	26 (36.60)	28 (39.40)	0.740
Hospitalization D	12.26 (14.56)	25.05 (22.96)	0.030	20.86 (26.22)	21.24 (16.78)	0.945
ICU admission (%)	4 (5.60)	19 (26.80)	0.013	8 (11.30)	15 (21.10)	0.090
MV (%)	1 (1.40)	7 (9.90)	0.114	2 (2.80)	6 (8.50)	0.145
Tracheostomy (%)	1 (1.40)	4 (5.60)	0.389	1 (1.40)	4 (5.60)	0.174
Comorbidity (%)	17 (23.90)	33 (46.50)	0.281	22 (31)	28 (39.40)	0.168
Charlson index	2.25 (1.07)	2.83 (1.44)	0.096	1.76 (0.69)	3.39 (1.31)	< 0.001
Dyspnea (%)	19 (26.80)	29 (40.80)	0.697	27 (38)	21 (19.60)	0.090
MW/fatigue (%)	27 (38)	44 (62)	0.250	35 (49.29)	36 (50.71)	

Data are provided as mean (standard deviation and percentage).

BMI, body mass index; Hospitalization D, Days of Hospitalization; ICU, intensive care unit; MV, mechanical ventilation; MW, muscle weakness; N, number of patients with COVID-19.

**Table 2:** Univariate and multivariate linear regression for ECW/TBW on descriptive, clinical and cardiorespiratory fitness variables.

	**Univariate**			**Multivariate**			
**Variables**	β **coef**.	**95% CI**	***p*** **-Value**	β **coef**.	**95% CI**	***p*** **-Value**	**VIF**
Age (years)	0.001	0.000 to 0.001	< 0.001	0.000	0.000–0.001	0.575	2.2
Sex	−3.87·10^−5^	−0.005 to 0.004	0.986				
^Log^BMI (kg·m^−2)^	0.010	−0.021 to 0.041	0.505				
Hospital admission	−2.29·10^−5^	−0.005 to 0.005	0.993				
Mechanical ventilation	0.005	−0.002 to 0.012	0.158				
Tracheostomy	0.010	0.002 to 0.018	0.020	0.002	−0.007 to 0.012	0.618	1.3
Dyspnea	0.000	−0.004 to 0.005	0.912				
MW/Fatigue	0.013	−0.001 to 0.026	0.062	−0.005	−0.017 to 0.006	0.365	1.3
Comorbidities	0.003	−0.002 to 0.007	0.284				
Charlson	0.004	0.003 to 0.006	< 0.001	0.003	0.001 to 0.005	0.002	1.9
^Log^VO_2peak_ (ml·kg^−1^·m^−1^)	−0.022	−0.036 to −0.007	0.004	−0.010	−0.020 to −0.000	0.048	3.6
VE·${\text{VCO}}_{2}^{-1}$ slope	0.000	0.000 to 0.000	0.445				
VE (L·m^−1^)	0.000	0.000 to 0.000	0.065	1.67·10^−6^	0.000 to 0.000	0.990	3.2
PetO_2_ (mmHg)	0.000	0.000 to 0.001	0.157				
PetCO_2_ (mmHg)	0.000	−0.001 to 0.000	0.087	0.000	−0.001 to 0.000	0.364	1.8
RER	0.001	−0.011 to 0.012	0.924				
Heart rate (beats·m^−1^)	−6.93·10^−5^	0.000 to 0.000	0.216				
Time trial (mm:ss)	−5.16·10^−5^	0.000 to 0.000	0.525				
Power output (W)^*^	−6.69·10^−5^	0.000 to 0.000	0.016				
O_2_ Saturation (%)	0.000	−0.002 to 0.001	0.744				

95% CI, 95% Confidence Interval; BMI, body mass index; coef., coefficient; ECW, extracellular water; Log, logarithmic transformation of the data; MW, muscular weakness; PetCO_2_, End-tidal CO_2_ pressure; PetO_2_, End-tidal O_2_ pressure; RER, respiratory exchange ratio; TBW, total body water; VE, minute ventilation; VCO_2_, carbon dioxide volume; VIF, variance inflation factors; peak VO_2_, peak oxygen uptake. Only variables with p < 0.10 in univariate analyses were considered in the multivariate model.

^*^Power output showed collinearity with peak VO_2_ in preliminary models and was excluded to improve model stability.

**Table 3:** Univariate and multivariate linear regression for ECW/TBW on descriptive, clinical and muscular fitness variables.

	**Univariate**			**Multivariate**			
**Variables**	β **coef**.	**95% CI**	***p*** **-Value**	β **coef**.	**95% CI**	***p*** **-Value**	**VIF**
Age (years)	0.001	0.000 to 0.001	< 0.001	0.000	−0.001 to 0.001	0.736	2.2
Tracheostomy	0.010	0.002 to 0.018	0.020	0.005	−0.006 to 0.015	0.381	1.4
Charlson index	0.004	0.003 to 0.006	< 0.001	0.004	0.002 to 0.006	< 0.001	2.1
FSST (s)	−1.77·10^−5^	0.000 to 0.000	0.924				
TUG test	0.001	0.000 to 0.002	0.121				
Sit-to-Stand test	0.000	−0.001 to 0.000	0.516				
**Peak torque (N**·**m)**							
60° Right knee ext.	−3.47·10^−5^	0.000 to 0.000	0.368				
60° Left knee ext.	−7.82·10^−5^	0.000 to 0.000	0.095	−7.56·10^−5^	0.000 to 0.000	0.070	1.1
180° Right knee ext.	−2.71·10^−5^	0.000 to 0.000	0.598				
180° Left knee ext.	−3.44·10^−5^	0.000 to 0.000	0.634				
60° Right knee flex.	−8.31·10^−5^	0.000 to 0.000	0.176				
60° Left knee flex.	0.000	0.000 to 0.000	0.138				
180° Right knee flex.^*^	0.000	−0.008 to 0.009	0.980				
180° Left knee flex.	−1.86·10^−5^	0.000 to 0.000	0.814				
**Power output (W)**							
60° Right knee ext.	−3.74·10^−5^	0.000 to 0.000	0.571				
60° Left knee ext.	0.000	0.000 to 0.000	0.143				
180° Right knee ext.^*^	−0.002	−0.014 to 0.010	0.735				
180° Left knee ext.	−5.21·10^−5^	0.000 to 0.000	0.434				
60° Right knee flex.	−9.61·10^−5^	0.000 to 0.000	0.260				
60° Left knee flex.	0.000	0.000 to 0.000	0.234				
180° Right knee flex.	−9.73·10^−5^	0.000 to 0.000	0.867				
180° Left knee flex.	1.76·10^−5^	0.000 to 0.000	0.821				

Descriptive and clinical variables are the same as in cardiorespiratory fitness.

95% CI, 95% Confidence Interval; coef., coefficient; Ext, extension; Flex, flexion; FSST, four square step test; TUG, Timed Up and Go; VIF, variance inflation factors.

^*^Logarithmic transformation of data. Only variables with p < 0.10 in univariate analyses were considered in the multivariate model.

### 3.1 Univariate analysis

Among CRF variables, VO_2_peak and power output showed inverse associations with ECW/TBW ratio (β = −0.022, 95% CI: −0.036 to −0.007, *p* = 0.004; β = −6.69·10^−5^, 95% CI: 0.000 to 0.000, *p* = 0.016; respectively; Table 2). Most MF variables were not associated with ECW/TBW; however, peak torque at 60° left knee extension met the predefined selection criterion (*p* < 0.10) and was therefore included in the multivariate model (Table 3).

### 3.2 Multivariate analysis

In the final CRF multivariable model, which included seven predictors, the Charlson Comorbidity Index and log-transformed VO_2_peak remained independently associated with ECW/TBW after multivariable adjustment (β = 0.003, 95% CI: 0.001–0.005, *p* = 0.002; β = −0.010, 95% CI: −0.020 to 0.000, *p* = 0.048, respectively). The model explained 44.6% of the variance in ECW/TBW (*R*^2^ = 0.446; adjusted *R*^2^ = 0.365). Multicollinearity diagnostics indicated acceptable VIF values for all predictors (VIF ≤ 3.6). Redundant exercise-derived variables (e.g., power output), which showed collinearity with VO_2_peak in preliminary models, were excluded to improve model stability. No statistically significant associations were observed for the remaining variables (*p* > 0.05; Table 2).

In the MF multivariate model, which included four predictors, the Charlson Comorbidity Index was the only variable that retained statistical significance (β = 0.004, 95% CI: 0.002–0.006, *p* < 0.001). This model explained 52.4% of the variance in ECW/TBW (*R*^2^ = 0.524; adjusted *R*^2^ = 0.464). Associations between ECW/TBW and MF variables did not reach statistical significance, with confidence intervals overlapping the null value, indicating limited evidence for independent associations in this sample. No relevant collinearity was observed in the MF model (all VIF values ≤ 2.2; Table 3).

### 3.3 ROC analysis

**Figure 2:**
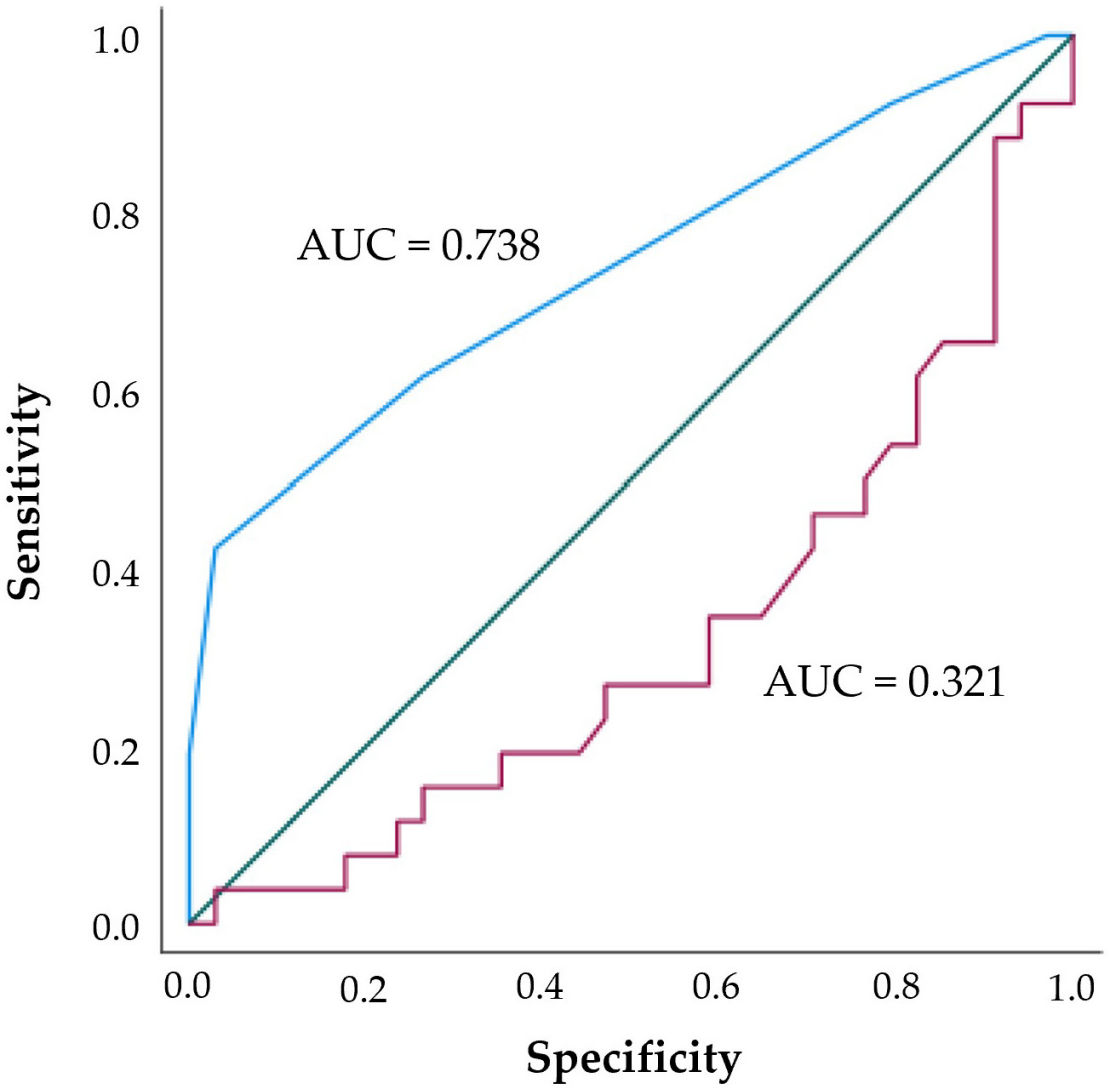
Receiver-operating curve (ROC) for the Charlson Comorbidity Index (blue line; AUC = 0.74, indicating good discrimination) and log-transformed relative VO_2_peak (red line; AUC = 0.32, indicating poor discrimination) in identifying participants with elevated ECW/TBW ratio (≥0.40). The green line represents the reference (no discrimination) line.

The ROC curve analysis showed that the Charlson Comorbidity Index had a good ability to discriminate between participants with elevated ECW/TBW ratios (≥0.40) and those with lower values. The AUC was 0.74 (95% CI: 0.61–0.87; *p* < 0.001), indicating good predictive performance. Using the optimal cut-off identified by the Youden index, a Charlson Comorbidity Index ≥3 showed a sensitivity of 61.5% and a specificity of 73.5% for identifying elevated ECW/TBW. In contrast, log-transformed VO_2_peak demonstrated poor discriminatory ability for the same outcome (AUC = 0.32; 95% CI: 0.18–0.46; *p* = 0.011), indicating an inverse and non-discriminatory relationship; therefore, no optimal cut-off with corresponding sensitivity and specificity was derived (Figure 2).

Table 4 presents the results of the comparative analyses by sex and age. Men showed significantly higher values for absolute VO_2_peak (*p* = 0.007), minute ventilation (VE; *p* < 0.001), time to exhaustion (*p* < 0.001), and power output during CPET (*p* < 0.001) compared to women. In the isokinetic evaluation, men exhibited greater peak torque in left knee extension at 60° (*p* = 0.037) and flexion at 60° (*p* = 0.043), as well as higher power output in left knee flexion at 60° (*p* = 0.045). No significant differences were observed in functional performance tests (FSST, TUG, 30-Second Sit-to-Stand; *p* > 0.05). When stratified by age (< 64 vs. ≥64 years), younger participants displayed significantly greater power output during CPET (*p* = 0.017).

**Table 4:** Differences in cardiorespiratory and muscular fitness variables by sex and age in older adults with post-COVID-19 syndrome (*n* = 71).

**Variable**	**Women**	**Men**	***p*-Value**	** < 64 years**	**>64 years**	***p*-Value**
**Cardiorespiratory fitness**						
VO_2peak_ (ml·kg^−1^·m^−1^)	13.30 (3.36)	17.47 (7.10)	0.007	17.09 (8.11)	14.74 (3.49)	0.217
VE·${\text{VCO}}_{2}^{-1}$ slope	34.23 (9.43)	34.03 (8.02)	0.928	32.95 (9.64)	35.26 (7.18)	0.266
VE (L·m^−1^)	40.89 (12.04)	58.05 (16.85)	< 0.001	53.94 (19.85)	49.30 (14.29)	0.268
PetO_2_ (mmHg)	114.96 (6.30)	113.88 (4.96)	0.433	113.65 (4.86)	114.91 (6.04)	0.341
PetCO_2_ (mmHg)	33.85 (4.92)	34.88 (6.20)	0.471	35.82 (5.95)	33.20 (5.30)	0.057
RER	1.26 (0.19)	1.28 (0.22)	0.700	1.28 (0.21)	1.27 (0.21)	0.896
Heart rate (bpm)	125.15 (20.14)	124.91 (20.78)	0.962	125.82 (23.85)	124.20 (6.68)	0.744
Time trial (mm:ss)	06:06 (01:35)	08:38 (02:13)	< 0.001	08:15 (02:38)	07:08 (01:55)	0.051
Power output (W)	72.62 (22.77)	105.84 (43.61)	< 0.001	104.97 (47.52)	82.00 (28.23)	0.017
O_2_ Saturation (%)	98.00 (1.29)	97.44 (1.70)	0.166	98.00 (1.23)	97.29 (1.80)	0.066
**Muscular fitness**						
FSST (s)	26.84 (6.78)	23.78 (7.21)	0.163	24.47 (8.56)	25.44 (5.11)	0.649
TUG test	7.43 (1.97)	7.06 (2.84)	0.635	6.75 (2.50)	7.73 (2.54)	0.194
Sit-to-Stand test	19.35 (3.26)	18.34 (5.47)	0.494	18.80 (5.83)	18.62 (3.17)	0.899
**Peak torque (N**·**m)**						
60° Right knee ext.	98.24 (36.57)	113.60 (29.86)	0.135	115.72 (30.03)	98.00 (34.70)	0.077
60° Left knee ext.	94.42 (19.61)	111.67 (29.08)	0.037	111.86 (23.15)	96.78 (29.42)	0.064
180° Right knee ext.	58.04 (30.94)	61.75 (20.88)	0.637	65.41 (27.49)	54.20 (20.57)	0.140
180° Left knee ext.	54.78 (18.36)	62.72 (16.90)	0.149	63.76 (17.11)	54.72 (17.54)	0.092
60° Right knee fle.	48.14 (25.57)	60.91 (16.38)	0.052	60.29 (21.45)	50.76 (20.20)	0.143
60° Left knee flex.	50.59 (20.20)	62.47 (16.93)	0.043	61.22 (20.52)	53.80 (16.68)	0.205
180° Right knee flex.	29.27 (27.73)	32.60 (17.14)	0.628	34.92 (24.11)	27.27 (17.57)	0.247
180° Left knee flex.	29.06 (19.97)	33.38 (14.34)	0.415	34.15 (18.12)	29.04 (14.53)	0.318
**Power output (W)**						
60° Right knee ext.	55.16 (22.74)	64.74 (16.31)	0.111	63.44 (19.31)	58.16 (19.56)	0.375
60° Left knee ext.	56.51 (15.48)	65.01 (16.02)	0.090	65.79 (15.69)	56.85 (15.76)	0.067
180° Right knee ext.	61.04 (37.12)	63.73 (26.61)	0.781	68.56 (35.21)	55.64 (23.16)	0.167
180° Left knee ext.	54.36 (16.02)	62.94 (20.57)	0.151	62.89 (20.05)	55.71 (17.87)	0.221
60° Right knee flex.	29.20 (19.21)	36.51 (11.61)	0.127	35.52 (16.27)	31.44 (14.18)	0.389
60° Left knee flex.	29.98 (13.80)	37.32 (9.47)	0.045	35.73 (13.51)	32.92 (9.57)	0.443
180° Right knee flex.	25.32 (27.67)	27.83 (19.44)	0.728	30.51 (25.62)	22.75 (18.19)	0.265
180° Left knee flex.	24.31 (18.94)	28.23 (15.56)	0.465	27.98 (18.88)	25.38 (14.35)	0.619

Data are provided as mean (standard deviation).

bpm, beats per minute; ext, extension; flex, flexion; FSST, four square step test; PetCO_2_, End-tidal CO_2_ pressure; PetO_2_, End-tidal O_2_ pressure; RER, respiratory exchange ratio; TUG, Timed Up and Go; val, value; VE, minute ventilation; VCO_2_, carbon dioxide volume; VO_2peak_, peak oxygen uptake; W, watts.

## 4 Discussion

This study examined the relationship between hydration status—specifically the extracellular water-to-total body water (ECW/TBW) ratio—and both CRF and MF in older adults with post-COVID-19 syndrome. The results showed that higher ECW/TBW ratio was significantly associated with lower VO_2_peak and greater comorbidity burden, while no significant associations were observed with MF variables. Among the predictors examined, the Charlson Comorbidity Index emerged as the strongest determinant of elevated ECW/TBW ratio. In addition, sex-related differences in CRF and selected lower-limb strength measures were more evident than those related to age. Taken together, these findings highlight that altered fluid distribution is more closely linked to systemic health status and aerobic capacity than to localized muscular function in this population.

The prominent role of the Charlson Comorbidity Index in the present study warrants specific consideration. Beyond its use as an adjustment variable, comorbidity burden emerged as the strongest and most consistent predictor of an elevated ECW/TBW ratio across multivariable models and demonstrated good discriminatory performance in ROC analyses. This finding suggests that ECW/TBW may, to a substantial extent, reflect the cumulative systemic impact of chronic comorbid conditions rather than isolated organ dysfunction. The Charlson Comorbidity Index is a well-validated summary measure of multimorbidity that has consistently demonstrated prognostic value across diverse clinical populations, with stepwise increases in the index associated with higher mortality risk and worse clinical outcomes (42, 43). In post-COVID-19 populations, higher Charlson scores have been linked to poorer clinical trajectories and increased vulnerability, supporting its role as an integrative indicator of systemic disease burden (44).

The strong association between the Charlson Comorbidity Index and a higher ECW/TBW ratio observed in our study has a plausible pathophysiological basis. Several of the main comorbidities weighted by the Charlson Comorbidity Index, such as heart failure, chronic kidney disease, and diabetes, are pro-inflammatory conditions characterized by endothelial dysfunction and hormonal dysregulation, which directly promote sodium retention and extracellular fluid expansion (50–53). Although these mechanisms were not directly assessed in the present study, they provide a biologically coherent framework to interpret the observed association between comorbidity burden and altered fluid distribution. This pre-existing vulnerability may be further exacerbated in the context of post-COVID-19 syndrome. SARS-CoV-2 infection has been shown to induce multiorgan involvement, persistent systemic inflammation, and dysregulation of the renin–angiotensin–aldosterone system, thereby contributing to altered fluid homeostasis (54).

Therefore, it is plausible that in our cohort of older adults with post-COVID-19 syndrome, the chronic comorbidity burden quantified by the Charlson Comorbidity Index acts as a key determinant of a subclinical overhydration state, as reflected by an elevated ECW/TBW ratio, which in turn is associated with reduced aerobic capacity. This interpretation is consistent with previous studies identifying the Charlson Comorbidity Index as a robust predictor of adverse outcomes across a wide range of clinical populations (42, 43), including post-COVID-19 populations (44). In this context, an elevated ECW/TBW ratio may represent an integrative marker of multisystem dysregulation driven by comorbidity load rather than a simple reflection of reduced physical fitness.

Importantly, the interpretation of these associations must be framed within the constraints of the cross-sectional design. The directionality of the relationships between ECW/TBW, comorbidity burden, and CRF cannot be established, and multiple causal pathways are plausible. Altered hydration status and fluid distribution may impair pulmonary diffusion and promote peripheral congestion, which in turn may adversely affect aerobic capacity (55, 56). Conversely, reduced CRF and physical deconditioning may promote adverse fluid distribution via metabolic and inflammatory mechanisms (34, 57). In this context, ECW/TBW should be viewed as a clinically meaningful biomarker reflecting hydration status, nutritional state, and systemic inflammatory burden—factors that play a central role in the pathophysiology of numerous chronic diseases (34). Furthermore, comorbidity burden may act as a common upstream determinant influencing both ECW/TBW and CRF, positioning ECW/TBW as a downstream manifestation or partial mediator rather than a direct causal factor. Accordingly, ECW/TBW is best interpreted as an integrative marker of systemic vulnerability rather than evidence of a unidirectional causal mechanism.

Evidence from previous research indicates that each one-unit increase in ECW/TBW beyond the inflection point is associated with a 10% higher risk of all-cause mortality, underscoring its potential value as a prognostic marker to guide targeted interventions (34). In parallel, VO_2_peak is a well-established prognostic indicator, consistently linked to all-cause and cardio-vascular mortality across diverse clinical cohorts (10). The present findings extend this evidence by showing that both parameters are interrelated in older adults with post-COVID-19 syndrome: elevated ECW/TBW was associated with greater comorbidity burden and lower VO_2_peak. This suggests that impaired fluid regulation and reduced aerobic capacity may act synergistically as markers of clinical vulnerability in older adults with post-COVID-19 syndrome, as occurs in other clinical settings (58, 59). Importantly, combining ECW/TBW assessment with VO_2_peak evaluation may enhance risk stratification beyond the use of either marker alone.

Mechanistically, excess extracellular fluids may compromise exercise tolerance through several interdependent pathways. At the pulmonary level, interstitial edema reduces alveolar–capillary membrane conductance and impairs gas diffusion, thereby limiting oxygen transfer during exertion (60, 61). Beyond the lungs, peripheral congestion further restricts oxygen delivery and utilization, while skeletal muscle mitochondrial dysfunction reduces contractile efficiency, both directly associated with lower VO_2_peak (62, 63). These pathophysiological processes are not merely theoretical but have been consistently documented in post-COVID-19 populations. Persistent systemic inflammation, endothelial dysfunction, and multiorgan sequelae—including cardiovascular and pulmonary complications—may exacerbate these impairments by disrupting fluid homeostasis and promoting extracellular water retention (64, 65). Residual interstitial changes, vascular involvement, and reduced diffusion capacity have been observed months after infection (66–68), further compromising pulmonary gas exchange and limiting exercise tolerance. Such findings are consistent with the reduced VO_2_peak observed in our study and emphasize the clinical relevance of jointly assessing fluid status and CRF in patients with post-COVID-19 syndrome (6, 13, 69).

Taken together, these results reinforce the clinical importance of the ECW/TBW ratio as both an indicator of fluid imbalance and a surrogate marker of overhydration—a condition consistently linked to adverse outcomes in hospitalized and chronic patient populations (30). When considered alongside VO_2_peak, which integrates cardiovascular, pulmonary, and muscular function, the ECW/TBW ratio provides complementary information that may enhance risk stratification and guide individualized rehabilitation strategies in older adults with post-COVID-19 syndrome.

In contrast, no significant associations were found between the ECW/TBW ratio and MF measures, including isokinetic strength and functional performance tests. This lack of correlation may reflect that extracellular fluid shifts do not directly affect localized muscle function, or that standard muscular assessments do not capture the nuanced complexity of fluid compartmentalization in older post-COVID-19 patients. Supporting this, Hioka et al. (33) reported that ECW/TBW acted as a confounding factor rather than a direct correlate of handgrip strength or gait speed in elderly women when using BIA for sarcopenia diagnosis. Moreover, accumulating evidence suggests that muscular strength and functional capacity are more closely related to intracellular water than to extracellular fluid distribution. For instance, Serra-Prat et al. (70) demonstrated significant associations between intracellular water and muscle strength as well as frailty status in community-dwelling older adults. Together, these findings support the notion that muscle function may be more strongly linked to intracellular hydration, muscle quality, and cellular integrity rather than to extracellular fluid balance, which may partly explain the absence of significant associations with ECW/TBW observed in our cohort. However, the present study may have been underpowered to detect weaker associations, and formal analyses of intracellular water were beyond the scope of this work.

In line with this reasoning, and given that muscular strength is closely related to muscle quality and cellular integrity, phase angle was additionally explored as a complementary bioelectrical marker (Supplementary Table S1). Phase angle has been widely interpreted as an indicator related to the cellular membrane integrity and has been linked to reduced muscle strength, impaired mobility, and lower levels of physical performance in older populations (71–73). Given this evidence, phase angle might reveal an association with MF where ECW/TBW did not. However, no significant association was observed between phase angle and MF in our sample. It was not possible to determine whether the lack of association between ECW/TBW and muscular strength reflects a genuine characteristic of the sample or a limitation of fluid-related bioelectrical markers in adequately capturing tissue quality. Therefore, non-significant results should not be interpreted as definitive evidence of no relationship, and further studies specifically designed to examine the role of fluid compartments in MF—particularly in post-COVID-19 populations—are warranted.

Sex-related differences deserve specific consideration when interpreting the associations between ECW/TBW and CRF and MF. In the present study, men exhibited significantly higher VO_2_peak, ventilation, power output, and several muscular strength measures compared with women, in line with well-established sex-related differences in aerobic capacity, strength, and muscle mass across adulthood and aging (74–76). These differences were evident despite similar age and clinical profiles, underscoring the physiological relevance of sex in functional performance.

Sex differences in body water distribution may further influence the interpretation of ECW/TBW values. Prior studies have demonstrated that total body water and its compartmentalization are strongly influenced by sex and body composition, with women exhibiting lower total body water per kilogram of body weight than men at comparable BMI levels, largely due to differences in fat-free mass (77). In addition, evidence suggests that the proportion of ECW/TBW differs between men and women and may increase with adiposity, although not always in a linear or intuitive manner (77, 78).

Importantly, at similar BMI levels, women do not consistently exhibit higher extracellular water proportions than men despite having greater fat mass, suggesting that factors beyond body composition—such as sex-specific regulation of fluid balance, hormonal influences, and renal sodium handling—may play a role (77–79). Moreover, methodological considerations related to bioelectrical impedance–derived equations, which have historically been developed predominantly in male populations, may also contribute to sex-specific differences in ECW/TBW estimation (77).

Although sex was not independently associated with ECW/TBW in univariate analyses and was therefore not retained in the final multivariable models, the marked sex differences observed in VO_2_peak suggest that sex may act as an effect modifier of the ECW/TBW–CRF relationship. Accordingly, ECW/TBW values should be interpreted within a sex-specific physiological context to avoid overgeneralization, particularly when relating hydration-related markers to functional outcomes in older adults with post-COVID-19 syndrome. Future studies with larger samples should explore formal interaction analyses to better characterize sex-specific patterns in the association between fluid distribution and aerobic capacity.

Our study has several strengths, including a well-characterized cohort of older adults with post-COVID-19 syndrome, the use of comprehensive CRF and MF assessments, and the application of advanced body composition analysis techniques. However, several limitations should be acknowledged. The cross-sectional design inherently limits causal inference, as the directionality of the associations between ECW/TBW, comorbidity burden, and physical fitness outcomes cannot be established. Consequently, it remains unclear whether an elevated ECW/TBW ratio acts as a causal contributor to impaired CRF, represents a consequence of reduced functional capacity and clinical status, or primarily reflects an underlying state of systemic vulnerability. It is plausible that multiple causal pathways coexist, with comorbidity burden acting as a common upstream determinant influencing both fluid distribution and functional capacity. Accordingly, ECW/TBW should be interpreted as an integrative biomarker of clinical vulnerability rather than evidence of a unidirectional causal mechanism.

Although the sample size was adequate for exploratory analyses, it may have limited the statistical power to detect weaker associations, particularly with MF variables, and restricts the generalizability of the findings. Furthermore, given the exploratory nature of the study and the sample size, the ability to detect modest associations—particularly between ECW/TBW and MF outcomes—may have been limited. Muscle function is influenced by multiple physiological dimensions that may not be fully captured by extracellular fluid-related markers alone. Therefore, the lack of statistically significant associations with MF should be interpreted cautiously and within the context of these methodological considerations.

In addition, the number of predictors initially considered—especially in the CRF model—raised the potential risk of overfitting and type I error due to multiple testing. To mitigate this risk, model complexity was reduced, redundant exercise-derived variables were excluded, and multicollinearity was formally assessed using variance inflation factors, resulting in stable final models with acceptable collinearity.

The study cohort consisted exclusively of post-COVID-19 patients recruited from a single center, which may further limit external validity and applicability to other clinical settings or community-dwelling older adults. Moreover, participants were evaluated in a rehabilitation setting, which may preferentially select individuals with a higher symptom burden or greater functional impairment, potentially limiting the generalizability of the findings to less symptomatic post-COVID-19 populations. Although the study focused on older adults, the age range was relatively narrow, with most participants in their early to mid-60s. Therefore, the present findings may not be generalizable to the oldest old (e.g., ≥80 years), to more frail populations, or to individuals with more advanced functional impairment.

Post–COVID-19 syndrome is characterized by a heterogeneous and potentially episodic clinical course, with periods of symptom remission and exacerbation. Given the cross-sectional design, the present study represents a single temporal snapshot of a dynamic condition. Although time since acute infection was recorded and reported, we could not account for symptom fluctuations or acute inflammatory flare-ups at the time of assessment, which may have influenced physiological outcomes sensitive to transient inflammatory processes. Therefore, a degree of temporal selection bias cannot be excluded, and longitudinal studies are needed to better characterize temporal trajectories.

Information on medication use, including drugs that may influence fluid balance and exercise performance (e.g., diuretics, beta-blockers, corticosteroids), was not systematically collected and therefore could not be included in the analyses. This omission may have resulted in residual confounding and should be considered when interpreting the findings.

Finally, bioelectrical impedance analysis, while practical and widely validated, is sensitive to hydration status and device-specific equations, which may introduce measurement variability despite the use of standardized assessment procedures. Nevertheless, the consistency of associations across models and the use of validated clinical and functional assessment tools strengthen the reliability of the present findings.

Future research should explore longitudinal changes in body water compartments and fitness parameters during and after rehabilitation programs in this population. Moreover, integrating biomarkers of inflammation and endothelial function could provide further insights into the mechanisms linking cardiorespiratory capacity, comorbidity burden, and body water regulation.

## 5 Conclusions

In this exploratory, cross-sectional study of older adults with post-COVID-19 syndrome, a higher ECW/TBW ratio was consistently associated with greater comorbidity burden and lower CRF, while no significant associations were observed with MF. The Charlson Comorbidity Index emerged as the strongest determinant of elevated ECW/TBW, supporting the interpretation of altered fluid distribution as a reflection of cumulative systemic disease burden rather than isolated functional impairment.

Notably, marked sex-related differences were observed across several CRF and MF parameters and were more pronounced than age-related differences. Although sex was not independently associated with ECW/TBW in multivariable models, these findings suggest that sex may modify the relationship between hydration-related markers and functional outcomes, underscoring the need for sex-specific interpretation of ECW/TBW values in older post-COVID-19 populations.

Given the cross-sectional and exploratory nature of the study, causal inferences cannot be established. ECW/TBW should therefore be interpreted primarily as an integrative marker of systemic vulnerability—closely linked to comorbidity burden and reduced aerobic capacity—rather than as a direct causal factor or a simple consequence of physical deconditioning.

Future longitudinal studies are warranted to clarify temporal relationships between fluid distribution, comorbidity burden, and functional decline, and to determine whether changes in ECW/TBW over time may help guide individualized rehabilitation and risk stratification strategies in older adults with post-COVID-19 syndrome.

## Data Availability

The raw data supporting the conclusions of this article will be made available by the authors, without undue reservation.
