# Supplementary material for: Extracellular-to-total body water ratio is associated with comorbidity and cardiorespiratory fitness in older adults with post-COVID-19 syndrome
Source: Front Nutr. 2026 Feb 11;13:1715783. doi: 10.3389/fnut.2026.1715783 (PMC12932222; doi:10.3389/fnut.2026.1715783)
Supplement: Supplementary file 1 [file Table_1.docx]

Supplementary Material. Univariate and multivariate linear regression for phase angle on descriptive, clinical and muscular fitness variables

|  | **Univariate** | | |  | **Multivariate** | | |  |
| --- | --- | --- | --- | --- | --- | --- | --- | --- |
| **Variables** | **β coef.** | **95% CI** | **p-value** |  | **β coef.** | **95% CI** | **p-value** | **VIF** |
| Age (years) | -0.074 | -0.108 to -0.039 | <0.001 |  | -0.030 | -0.078 to 0.017 | 0.208 | 2.1 |
| Sex | -0.393 | -0.782 to 0.003 | 0.048 |  | -0.521 | -0.888 to -0.153 | 0.006 | 1.2 |
| Mechanical ventilation | -0.929 | -1.502 to -0.356 | 0.002 |  | -0.392 | -1.364 to 0.581 | 0.423 | 2.6 |
| Tracheostomy | -1.401 | -2.082 to -0.719 | <0.001 |  | -0.404 | -1.659 to 0.850 | 0.521 | 2.7 |
| Charlson | -0.249 | -0.391 to -0.108 | <0.001 |  | -0.171 | -0.354 to 0.012 | 0.067 | 2.1 |
| FSST (s) | -0.002 | -0.035 to 0.031 | 0.915 |  |  |  |  |  |
| TUG test | -0.014 | -0.107 to 0.079 | 0.760 |  |  |  |  |  |
| Sit-to-Stand test | -0.015 | -0.064 to 0.035 | 0.555 |  |  |  |  |  |
| Peak torque (N·m) |  |  |  |  |  |  |  |  |
| 60º Right knee ext. | 0.005 | -0.003 to 0.012 | 0.218 |  |  |  |  |  |
| 60º Left knee ext. | 0.007 | -0.002 to 0.016 | 0.102 |  |  |  |  |  |
| 180º Right knee ext. | 0.002 | -0.008 to 0.012 | 0.700 |  |  |  |  |  |
| 180º Left knee ext. | 0.000 | -0.014 to 0.013 | 0.947 |  |  |  |  |  |
| 60º Right knee flex. | 0.004 | -0.008 to 0.016 | 0.512 |  |  |  |  |  |
| 60º Left knee flex. | 0.005 | -0.008 to 0.018 | 0.445 |  |  |  |  |  |
| *180º Right knee flex. | -0.002 | -0.014 to 0.009 | 0.684 |  |  |  |  |  |
| 180º Left knee flex. | -0.007 | -0.022 to 0.008 | 0.348 |  |  |  |  |  |
| Power output (W) |  |  |  |  |  |  |  |  |
| 60º Right knee ext. | 0.003 | -0.009 to 0.016 | 0.607 |  |  |  |  |  |
| 60º Left knee ext. | 0.007 | -0.008 to 0.022 | 0.329 |  |  |  |  |  |
| *180º Right knee ext. | 0.001 | -0.007 to 0.009 | 0.747 |  |  |  |  |  |
| 180º Left knee ext. | -0.001 | -0.013 to 0.012 | 0.917 |  |  |  |  |  |
| 60º Right knee flex. | 3.487·10^−5^ | -0.016 to 0.017 | 0.997 |  |  |  |  |  |
| 60º Left knee flex. | 0.001 | -0.020 to 0.022 | 0.923 |  |  |  |  |  |
| 180º Right knee flex. | -0.003 | -0.014 to 0.008 | 0.626 |  |  |  |  |  |
| 180º Left knee flex. | -0.009 | -0.024 to 0.006 | 0.236 |  |  |  |  |  |

Descriptive and clinical variables are the same as in cardiorespiratory fitness. Abbreviations: 95% CI = 95% Confidence Interval; coef. = coefficient; Ext = extension; Flex = flexion; FSST = four square step test; TUG = Timed Up and Go; VIF = variance inflation factors. *Logarithmic transformation of data. Only variables with p < 0.10 in univariate analyses were considered in the multivariate model
